# Evaluation of microcrack formation during root canal preparation using hand, rotary files and self-adjusting file in primary teeth: An in vitro study

**DOI:** 10.34172/joddd.2021.007

**Published:** 2021-02-13

**Authors:** Anup Panda, Krishna Shah, Varsha Budakoti, Krishna Dere, Mira Virda, Jina Jani

**Affiliations:** Department of Pediatric and Preventive Dentistry, College of Dental Sciences and Research Centre, Bopal, Manipur, Gujarat

**Keywords:** Dentinal microcracks, Primary teeth, Rotary files, SAF

## Abstract

**Background.** Pediatric endodontics is a field with constant evolution, resulting in the shifting of paradigms from the use of conventional hand files to rotary files for biomechanical preparation in primary teeth. Biomechanical preparation plays a crucial role in the success of root canal treatment. Primary teeth need special attention since they differ from permanent teeth in root canal morphology. Cleaning and shaping of the canals damage the root dentin, leading to dentinal cracks. Newer techniques for root canal preparation, including Ni-Ti rotary files and SAF system, have been developed for use in pediatric endodontics to overcome the drawbacks of conventional methods. The present study compared dentinal defects formed by rotary systems in primary teeth.

**Methods.** Eighty primary teeth were included. The teeth were decoronated with a diamond disc. All the roots were inspected for any pre-existing cracks or craze lines under transmitted light under a stereomicroscope. The specimens were then divided into four groups (n=20): group 1: control, group 2: hand files (HF), group 3: ProTaper files, and group IV: SAF files. The samples were instrumented according to the group they were assigned to.

**Results.** The HF and SAF groups exhibited fewer microcracks. Dentinal microcracks were observed in roots prepared with rotary ProTaper files. There were significant differences between HF/SAF and rotary files (*P* <0.05).

**Conclusion.** Stainless steel hand K-files and SAF instruments resulted in fewer dentinal damage than the ProTaper Universal files. SAF exhibited satisfactory results with minimal or no crack formation.

## Introduction

Endodontic techniques represent a fundamental step in the multidisciplinary approach of pediatric dentistry. Pulpal therapy of primary teeth is frequently performed to preserve the function and integrity of teeth in the dental arch. Endodontic therapy in deciduous teeth can be challenging and time-consuming, especially during cleaning and shaping the root canal, which is one of the most important steps in root canal treatment.^1^ Biomechanical preparation is one of the crucial steps that involves removing pathogenic bacteria and debris from the canal to achieve a successful treatment.^2^ Sometimes, biomechanical preparation of the canals damages the radicular dentin, leading to dentinal cracks and minute intricate fractures or even vertical root fracture and treatment failure.^3^ With advances in endodontic procedures, the incidence of damage to root dentin has also increased. Dentin undergoes structural damage following root canal preparation. Complexities in root canal anatomy and preparation are attributed to variations in the design of cutting instruments, taper, and composition of the material from which they are made.^4^ Stainless steel hand files are extensively used for the preparation of primary teeth and young permanent teeth initially.^5^ All the stainless-steel files tend to create aberrations because of the inherent stiffness of metal, confounded by the instrument design and root canal shape. Mostly, when stainless steel files are used in narrow curved canals, they restrict apical enlargement, hampering obturation.^6,7^

To resolve the difficulties with the use of stainless-steel instruments, Ni-Ti instruments were developed for curved canals without causing aberrations.^5^ Barrfirst describedthe use of Ni-Ti rotary files in primary root canals.^8^ These instruments exhibit enhanced flexibility and superior resistance to torsion fracture. In the last decades, the advent of Ni-Ti rotary instrumentation has transformed the endodontic treatment by minimizing the operator fatigue and the time required to complete the procedure, decreasing the chances of procedural errors compared with hand instrumentation.^9^

Considerable evidence is available in the literature showing that root canal preparation with Ni-Ti instruments in permanent teeth is efficient and effective. Similar principles can be applied for canal debridement and dentin shaping using Ni-Ti files in primary teeth. Ni-Ti files possess built-in features that help produce a predefined tapered shape in the canal. All the engine-driven file systems create many microcracks in the root dentin, making it more susceptible to vertical root fractures.

With the emergence of the self-adjusting file (SAF) System, the definition of “possible” in “as complete a job as possible” has markedly changed. After several research and development efforts, The SAF, developed by ReDent Nova, was introduced in 2010. This new technology uses a hollow, compressible Ni-Ti instrument without any central metal core. This hollow design helps in the continuous flow of endodontic irrigants throughout the procedure. The SAF technology uses a novel concept of canal debridement and shaping in which a uniform layer of radicular dentin is removed from the entire length of the canal, avoiding any excessive removal of sound dentin. Furthermore, unlike other rotary file systems, this file system does not apply machining of root canals to a circular bore, minimizing the risk of microcrack formation in the root dentin.^10^

Since there is no data in the literature on the comparative evaluation of the effect of rotary systems (Ni-Ti files), ProTaper Universal, SAF, and hand files on the incidence of root microcracks in primary teeth, this study might help understand the damage caused within the canal wall at different horizontal cross-sectional levels after root canal shaping with these files.

This in vitro study aimed to compare the incidence of cracks observed in the root dentin after root canal preparation with three different file systems in primary teeth.

The following file systems used:

1. K-files (Dentsply-Maillefer, Ballaigues, Switzerland)
2. ProTaper Universal files Sx, S1, S2, and F1 (Dentsply/Tulsa Dental, Tulsa, USA)
3. SAF system, 2 mm in thickness, #21 (ReDent-Nova, Raanana, Israel)

## Methods

Eighty single/multi-rooted extracted primary teeth with a minimum root length of 9 mm were included in the present in vitro study. The teeth were stored in distilled water. The teeth were extracted due to non-restorable crowns, over-retention of the teeth, and interventional orthodontics. Teeth with internal or external resorption, fractures, or root caries were excluded. The external surfaces of all the teeth were screened for any pre-existing cracks or craze lines by transmitted light under a stereomicroscope.

### Cleaning and shaping

The coronal portion of all the teeth was separated with a diamond disc under water cooling, leaving approximately 9‒10 mm of the root length. The samples were randomly divided into four groups according to file types used for biomechanical preparation of the root canal.

- Group 1: Untreated (control group)
- Group 2: Biomechanical preparation with hand files (K-files)
- Group 3: Biomechanical preparation with rotary ProTaper Universal files
- Group 4: Biomechanical preparation with the SAF system

A minimum sample size of 20 was required in each group. In all the teeth, apical patency was established by introducing a #15 K-file (Mani, Japan) into the root canal until its tip was visible at the apical foramen, and the working length (WL) was set 1.0 mm shorter than this measurement. Each instrument was replaced after preparing three teeth. Copious irrigation was carried out with 3% sodium hypochlorite and normal saline solutions between instruments using a syringe and a 27-gauge needle. At the end of the procedure, all the root canals were thoroughly cleansed with 2 mL of distilled water. All the roots were kept moist throughout the procedure.

Group 1 was left untreated and served as a control group. In the remaining groups, cleaning and shaping of the root canals were carried out according to the manufacturers’ instructions for each instrument system.

In group 2, the root canals were prepared manually using K-files (Mani Co, Tokyo, Japan). Instrumentation was carried out up to file #25 in the apical third, and then the step-back technique was followed to prepare the middle and coronal thirds of the canal. The root canals were irrigated with 2 mL of 3% NaOCl between instruments, followed by rinsing with 10 mL of 3% NaOCl and 10 mL of distilled water after completing the procedure.

In group 3, the biomechanical preparation was carried out with ProTaper rotary files (Dentsply- Maillefer, Ballaigues, Switzerland). Initially, a glide path preparation was carried out up to the WL using #15 and #20 K-files. ProTaper files were then used in the following sequence: S1 file was used to shape the root canal’s coronal third. The middle third was prepared with S2, and F1 was operated at the WL. Irrigation was carried out with 2 mL of 3% NaOCl after every instrument and 10 mL of 3% NaOCl; 10 mL of distilled water was used for flushing the canals at the end of the procedure.

In group 4, the biomechanical preparation was carried out with SAF. A glide path was prepared using #15 and #20 K-files. Cleaning and shaping of all the samples were carried out using the SAF system, #21 with a thickness of 2 mm (ReDentNova, Raanana, Israel) following the manufacturer’s recommendations. A Kavo Gentle low-speed handpiece (Kaltenbach & Voigt GmbH, Biberach, Germany) was connected to the RDT3 head (ReDent Nova, Ra’anana, Israel) at 5000 rpm and an amplitude of 0.4 mm. The canals were continuously irrigated with 3% NaOCl throughout the procedure at a rate of 5 mL/min for 4 minutes using a VATEA irrigation device (VATEA, ReDent, Ra’anana, Israel) incorporated with the SAF system.

All the roots were sectioned perpendicular to their long axis at 6, 4, and 2 mm using a diamond disc under water cooling. A digital camera (Olympus, Tokyo, Japan) connected to the stereomicroscope was used to capture images of each sectioned root at ×40 magnification.

**Figure 1:**
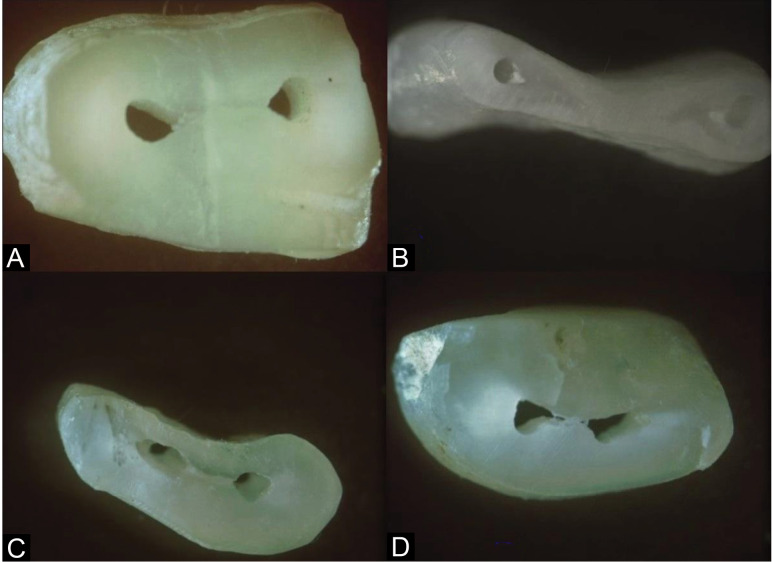
A- No defects, B- Craze lines, C- Vertical fracture D- Incomplete fracture.

Each specimen was assessed for the presence of any dentinal defects. Scoring was carried out by assessing the type of dentinal damage (Table 1 and Figure 1).

**Table 1:** Assessment of dentinal damage

**1**	No defect
**2**	Craze lines
**3**	Vertical fracture
**4**	Incomplete fracture

Score 1 (no defects) was assigned when radicular dentin was devoid of any lines or cracks in both the root canal’s external and internal surfaces. Score 2 (Craze lines) was assigned when lines were observed on the slice, extending from the outer surface towards the dentin or from the canal lumen to the dentin. Score 3 was assigned for vertical fractures. Score 4 (incomplete fracture); a separate entity of “fracture” was defined when the line extended from the root canal lumen to the root’s external surface. The results were tabulated by counting the number of roots with the defects in each group.

## Results

The data were analyzed with SPSS 17.0 (SPSS Inc., Chicago). A Pearson’s chi-square test was performed to determine statistically significant differences in defective roots’ appearance between the experimental groups. The level of significance was set at *P*< 0.05.

### Comparison between the groups

Tables 2, 3, and 4 present the frequencies and percentages of the defects. No defects were seen in the control group. In the apical third (6 mm), the prevalence of the defects was maximum in group 3 (n = 10), while group 2 exhibited fewer defects (n = 2), and group 4 had the least dentinal damage (n = 1).

**Table 2:** Comparison of defects and fractures in the apical third (n = 20)

	**No Defect**		**Fracture**		**Craze line**		**Incomplete Fracture**	
	**No.**	**%**	**No.**	**%**	**No.**	**%**	**No.**	**%**
Group 1	20	100	0	00	0	00	0	00
Group 2	18	90	0	00	2	10	0	00
Group 3	10	50	2	10	7	35	1	5
Group 4	19	95	0	00	1	5	0	00

**Table 3:** Comparison of defects and fractures in the middle third (n = 20)

	**No Defect**		**Fracture**		**Craze line**		**Incomplete Fracture**	
	**No.**	**%**	**No.**	**%**	**No.**	**%**	**No.**	**%**
Group 1	20	100	0	00	0	00	0	00
Group 2	16	80	0	00	3	15	1	5
Group 3	10	50	2	10	7	35	1	5
Group 4	19	95	0	00	1	5	0	00

**Table 4:** Comparison of defects and fractures in the cervical third (n = 20)

	**No Defect**		**Fracture**		**Craze line**		**Incomplete Fracture**	
	**No.**	**%**	**No.**	**%**	**No.**	**%**	**No.**	**%**
Group 1	20	100	00	00	00	00	00	00
Group 2	19	95	00	00	01	5	00	00
Group 3	14	70	01	5	04	20	01	5
Group 4	20	100	00	00	00	5	00	00

In the middle third (4 mm), again, the maximum number of defects was seen in group 3 (n = 10), while group 2 exhibited fewer defects (n = 4), and group 3had the least (n = 1), with no defects in the control group.

In the cervical third (2 mm), group 3 exhibited the maximum number of defects (n = 6), while group 2 showed the least (n = 1), and groups 1 and 4 had no defects (n = 0).

Comparison of the defects between the three groups showed that group 4 had the maximum number of no defects (Table 5).

**Table 5:** Comparison of no defects in between three group

		**Group**			**Total**	***P*** ** value**
		**H File**	**ProTaper**	**SAF**		
2 mm	Number	19	14	20	53	0.557
	% Within V1	35.8	26.4	37.7	100.0	
	% Within group	35.8	41.2	34.5	36.6	
4 mm	Number	16	10	19	45	0.247
	% Within V1	35.6	22.2	42.2	100.0	
	% Within group	30.2	29.4	32.8	31.0	
6 mm	Number	18	10	19	47	0.212
	% Within V1	38.3	21.3	40.4	100.0	
	% Within group	34.0	29.4	32.8	32.4	
Total	Number	53	34	58	145	
	% Within V1	36.6	23.4	40.0	100.0	
	% Within group	100.0	100.0	100.0	100.0	

Comparison of craze lines between different groups showed that the ProTaper group had the highest number of defects, which was statistically significant (*P*< 0.05) in the middle and apical thirds (Table 6). Vertical fractures were seen only in the ProTaper group (Table 7). Incomplete fractures were observed in groups 2 and 3, but the difference was not statistically significant (Table 8).

**Table 6:** Comparison of craze line in between three group

		**Group**			**Total**	***P*** ** value**
		**H File**	**ProTaper**	**SAF**		
2 mm	Number	1	4	0	5	0.070
	% Within V1	20.0%	80.0%	0.0%	100.0%	
	% Within group	16.7%	22.2%	0.0%	19.2%	
4 mm	Number	3	7	1	11	0.045
	% Within V1	27.3%	63.6%	9.1%	100.0%	
	% Within group	50.0%	38.9%	50.0%	42.3%	
6 mm	Number	3	7	1	11	0.045
	% Within V1	20.0%	70.0%	10.0%	100.0%	
	% Within group	33.3%	38.9%	50.0%	38.5%	
Total	Number	7	18	2	26	
	% Within V1	23.1%	69.2%	7.7%	100.0%	
	% Within group	100.0%	100.0%	100.0%	100.0%	

**Table 7:** Vertical fracture in the ProTaper group

		**Group**	**Total**
		**ProTaper**	
4 mm	Number	2	2
	% Within V1	100.0	100.0
	% Within group	50.0	50.0
6 mm	Number	2	2
	% Within V1	100.0	100.0
	% Within group	50.0	50.0
Total	Number	4	4
	% Within V1	100.0	100.0
	% Within group	100.0	100.0

**Table 8:** Comparison of incomplete fractures between hand & ProTaper groups

		**Group**		**Total**	***P*** ** value**
		**H file**	**ProTaper**		
2 mm	Number	0	2	2	0.157
	% Within V1	0.0	100.0	100.0	
	% Within group	0.0	50.0	40.0	
4 mm	Number	1	1	2	1.000
	% Within V1	50.0	50.0	100.0	
	% Within group	100.0	25.0	40.0	
6 mm	Number	0	1	1	0.317
	% Within V1	0.0	100.0	100.0	
	% Within group	0.0	25.0	20.0	
Total	Count	1	4	5	
	% Within V1	20.0	80.0	100.0	
	% Within group	100.0	100.0	100.0	

## Discussion

Preparation of the root canal system has been considered the most important step in endodontic therapy for dentin removal.^11^ Residual dentin thickness is the measure of mechanical limits of instrumentation to enlarge the canal diameter to approximately predetermined values that would not significantly weaken the dentinal walls.^12^ After completion of all the intra-radicular procedures, at least 1 mm of sound radicular dentin should remain in all aspects of the root along its entire length.^13^ Excessive removal of radicular dentin might result in strip perforation and vertical root fractures, especially in “danger zones.”^14^ The importance of retaining primary teeth until their successors’ eruption makes endodontic treatment the most common procedure in primary teeth.

The success of endodontic treatment depends upon the complete removal of necrotic tissue and proper sterilization of the root canal; therefore, biomechanical preparation of the root canal system is considered the most important aspect of endodontic treatment in primary teeth.^15,16^

Biomechanical preparation in primary teeth differs from that of permanent ones due to variations in their morphology because the roots of primary molars are more divergent and curved, allowing proper development of succedaneous teeth.^16,17^ These curvatures increase the incidence of perforation of the apical third of the root or the coronal one-third of the canal into the furcation.^15-18^ Additionally, excessive enlargement of the canal can reduce the dentinal thickness and weakens the tooth structure.^16-18^ It is important for clinicians to increase their knowledge of root canal anatomy because of the varied thickness of root dentin in different areas of teeth. The dentinal wall thickness is directly proportional to the ability of the tooth to withstand lateral forces. The primary objectives of preparing a root canal include the use of chemomechanical methods to remove the organic tissue and three-dimensional shaping of the canal system to achieve a continuously tapering preparation while maintaining the original outline of the canal.^19,20^

Root canal preparation using rotary files produces a variable degree of rotational force on the walls, creating microcracks or craze lines. The extent of these defects depends upon various precipitating factors, like constant or progressive taper, constant or variable pitch, cross-sectional geometry, tip design, and flute form of the instrument.^21^ Therefore, the present study was conducted on primary teeth to evaluate dentinal microcracks during biomechanical preparation. The tooth samples were sectioned with a diamond disc. No microcracks were observed in group 1, suggesting that all the cracks were due to root canal preparation and not by the sectioning method used. Microcracks were evaluated by digital cameras at magniﬁcations of ×10, ×12, ×20, and ×40.^21-24^

In the present study, a modified sequence was used for biomechanical preparation with ProTaper rotary files instead of hybrid or single length technique as there are no clear recommendations and guidelines for the instrumentation in primary teeth. Some researchers use the same recommendation as that of the permanent teeth, but others recommend modified methods. Due to the anatomical variations, like less dentinal thickness and density in primary teeth than permanent teeth, less mechanical preparation is advisable during their instrumentation. Therefore, the modified sequence was taken into consideration.^25^

In the present study, the frequencies of defects in the radicular dentin were 23.1%, 69.2%,and 7.7%with hand, ProTaper, and SAFs, respectively. Group 3 (SAFs) exhibited the lowest incidence of defects (7.7%), whereas the ProTaper group showed the maximum incidence of defects (69.2%) compared to other groups. Yoldas et al^21^ observed that rotary files induced the maximum number of defects (12/20). Hand instrumentation works by less aggressive movements in the root canal compared with the engine-driven instruments. Additionally, avoidance of continuous rotational movements and 2% less taper than Ni-Ti rotary files makes them safer for dentin.

In the present study, ProTaper Universal instruments were associated with a maximum number of microcracks, which might be attributed to the continuous, active rotational movements of the files in the root canal. According to Kim et al,^27^ whenever the taper of the files increases, stress concentration on the canal wall increases, resulting in dentinal damage, showing that progressive increase in the taper of these ProTaper files might contribute to the microcracks. Additionally, ProTaper universal files have a convex triangle shape in a transverse plane, which reduces the efflux of debris during the shaping of the canal, and these files have no radial area, which increases its deviation from the center of the canal, resulting in more stress towards the wall and creating microcracks.^28^

Minimal microcracks were observed in the SAF group as the SAFs work in the back-and-forth motion, which removes the dentin in scraping motion, unlike all other rotary systems which work in rotational motion. Additionally, these files do not have a cutting edge and flutes. This file system has a compressible and expansive lattice structure, which helps it to maintain intimate contact with the canal and then attempt to regain its original dimensions; thus, constant delicate pressure is applied on the canal walls, resulting in the uniform removal of dentin along the whole perimeter of the canal cross-section.^10^ Furthermore, these are hollow files, allowing continuous irrigation through the file, lubricating the canal and minimizing the generation of frictional stresses. These findings were consistent with several previous studies.^21,23,29^ indicating minimum dentinal damage while using SAF.^21,23,29^ Metzger^10^ suggested that SAF creates minimal stress of around 10 MPa on the dentin, which is significantly less than the tensile strength of dentin.^10^

## Conclusion

The present in vitro study compared the incidence of crack formation in the root dentin after biomechanical preparation, using three different instrument systems, ProTaper Universal, hand K-file, and SAFs in primary teeth. Within the limitations of the current study, the following conclusions can be drawn:

The control group where no biomechanical preparation was carried out showed no fractures. Biomechanical preparation tends to damage the root dentin. In comparison, it was observed that stainless steel hand K-files and SAF instruments exhibited less dentinal damage compared to the ProTaper Universal. SAF exhibited satisfactory results with minimal or no crack formation. This file system proved a better system than other rotary files as it caused less dentinal defects, decreasing the susceptibility to vertical root fracture.

It was also concluded that maximum fractures were seen in the middle third, followed by the apical third, with no fractures in the cervical third of the primary teeth.

## Authors’ Contributions

The present study was designed and supervised by AP. KS collected the specimens and performed the procedure. Each specimen was thoroughly checked and analyzed by VB and JJ. Data tabulation, statistical analysis and result interpretation were carried out by KD and MV. AP and VB contributed substantially to the discussion and drafted the manuscript. All authors critically revised the manuscript and gave final approval for publication.

## Acknowledgments

We thank all our friends, family members, and staff members for their constant support and encouragement to complete this study.

## Funding

The present study was sponsored by authors. No external funding from any individual or institutions was received by the authors.

## Competing Interests

The authors declare no competing interests with regards to authorship and /or publication of this article.

## Ethics Approval

Not applicable.
